# Impact of septic cerebral embolism on prognosis and therapeutic strategies of infective endocarditis: a retrospective study in a surgical centre

**DOI:** 10.1186/s12879-022-07533-w

**Published:** 2022-06-17

**Authors:** Valentina Scheggi, Silvia Menale, Barbara Tonietti, Costanza Bigiarini, Jacopo Giovacchini, Stefano Del Pace, Nicola Zoppetti, Bruno Alterini, Pier Luigi Stefàno, Niccolò Marchionni

**Affiliations:** 1 Division of Cardiovascular and Perioperative Medicine, Florence, Italy; 2 Division of General Cardiology, Florence, Italy; 3 Division of Cardiac Surgery, Florence, Italy; 4 Institute of Applied Physics “Nello Carrara” (IFAC), National Research Council, Florence, Italy; 5 Cardiothoracovascular Department, Azienda Ospedaliero-Universitaria Careggi and University of Florence, Florence, Italy; 6 Health Management Direction, Azienda Ospedaliero-Universitaria Careggi and University of Florence, Florence, Italy

**Keywords:** Endocarditis, Mortality, Prognosis, Infective endocarditis, Cerebral embolism, Outcome, Surgery

## Abstract

**Background:**

Infective endocarditis still has high mortality and invalidating complications, such as cerebral embolism. The best strategies to prevent and manage neurologic complications remain uncertain. This study aimed to identify predictors of cerebral septic embolism and evaluate the role of surgery in these patients in a real-world surgical centre.

**Methods:**

We retrospectively analyzed 551 consecutive patients admitted to our department with a definite diagnosis of non-device-related infective endocarditis; of these, 126 (23%) presented a neurologic complication.

**Results:**

Cerebral embolism was significantly more frequent in patients with large vegetations (p = 0.004), mitral valve infection (p = 0.001), and Staphylococcus aureus infection (p = 0.025). At multivariable analysis, only vegetation length was an independent predictor of cerebral embolism (HR per unit 1.057, 95% CI 1.025–1.091, p 0.001), with a best predictive threshold of 10 mm at ROC curve analysis (AUC 0.54, p = 0.001). Patients with neurologic complications were more often excluded from surgery despite an indication to it (16% vs 8%, p = 0.001). If eligible, they were treated within two weeks from diagnosis in similar proportions as patients without cerebral embolism with a similar survival rate. Predictors of mortality were hemorrhagic lesions (p = 0.018), a GCS < 14 (p = 0.001) or a severe degree of disability (p = 0.001) at presentation. The latter was the only independent predictor of mortality at multivariable analysis (HR 2.3, 95% CI 1.43–3.80, p = 0.001).

**Conclusions:**

The present study highlights the prognostic value of functional presentation and the safety of cardiac surgery, when feasible, in patients with cerebral septic embolism.

## Background

Despite recent diagnostic and therapeutic advances, the mortality rate of infective endocarditis (IE) still exceeds 20% in-hospital [1] and 30% at 3 years [2]. Neurologic events complicate 10–40% of left-sided IE and include embolic cerebrovascular complications, intracranial haemorrhage, ruptured mycotic aneurysm, transient ischaemic attack, meningitis, encephalopathy and brain abscess [3]. After a stroke, cardiac surgery should not be delayed in the absence of coma and after cerebral haemorrhage has been ruled out by CT [4]. Although heparinization during cardiac surgery has been supposed to exacerbate neurological deficits for secondary cerebral haemorrhage, previous studies showed that surgery is safe within 14 days of cerebrovascular event onset [5]. The optimal timing for operation is still debated and depends on the type of neurological complication and the urgency of valve replacement [3]. Moreover, prognostic factors in IE patients with neurologic events remain poorly investigated.

## Methods

### Patient selection

We have built a local registry of patients affected by non-device-related IE, where we recorded the 551 incident cases admitted to our department from January 2013 to November 2021. Device-related IE was defined as an infection on cardiac devices other than valve prosthesis. Data for analysis were retrieved from electronic hospital charts and were fully anonymized. The local Ethics Committee (Regional Ethics Committee of Tuscany for Experimental Medicine, Section: AREA VASTA CENTRO, n 12113_oss) approved the study and, in accordance with Italian laws for observational studies, granted a waiver of informed consent from study participants. We followed the current international IE guidelines for diagnostic work-up and treatment strategies and all methods were performed in accordance with them [4]. Brain CT or MR were performed in 352 (64%) patients, either for neurologic symptoms (89) or for the screening of embolism in asymptomatic patients (263). In patients with cerebral embolism (126), we reported the site of embolism, the presence of CT-detected hemorrhagic lesions, the Glasgow Coma Scale (GCS) and the Barthel index [6] determined immediately after the onset of the neurologic event. For the purpose of analysis, we categorized the GCS into two groups (15 or ≤ 14) and the Barthel index into three groups, indicating a substantially preserved overall functional independence (≥ 65), as opposed to a moderate (40–64) or severe (0–39) disability. Renal failure was defined as GFR < 60 mL/min/1.73 m^2^ (Mild GFR 45–59; Moderate GFR 30–44; Severe GFR 15–29). Multivariable analysis of all-cause in-hospital and long-term mortality was adjusted for the treatment received: surgery, medical therapy for the absence of surgical indication or exclusion from surgery for prohibitive clinical conditions. Age, gender, history of drug abuse, the microbiologic agent involved, left ventricular ejection fraction, type of valve (native or prosthetic) affected, double valve infection, the paravalvular extension of infection, severe valvular dysfunction, vegetation length, EUROSCORE II, CT-detected haemorrhagic complication of cerebral ischemic lesion, as well as the GCS and the Barthel index, were all entered into an initial multivariable Cox proportional hazards model.

### Follow-up

We calculated the follow-up duration from the time of IE diagnosis. A structured phone interview updated the follow-up of all patients to March 2022.

### Study endpoints

Identification of predictors of cerebral embolism and mortality in patients with neurologic complications of IE were the primary study endpoints.

### Statistical analysis

We used the chi-square and the Mann–Whitney or Kruskal–Wallis tests to compare respectively proportions and continuous variables with normal or non-normal distribution. We performed univariable and multivariable analyses using logistic regression and general linear models. We used the Kaplan–Meier method to estimate the univariate survival analysis and the Cox regression to identify the multivariable associations with mortality and estimate their hazard ratio with 95% confidence interval. All tests were 2-sided, and statistical significance was defined as a p-value < 0.05. We performed the analyses with SPSS 23.0 and R 3.6.3.

## Results

### Patient characteristics

Of 551 patients with IE in the registry, 126 (23%) had a neurologic complication. The median follow-up was 3.4 years (95% CI 3.2–3.6). The main demographic, clinical, echocardiographic and microbiologic characteristics of the cohort by the presence or absence of cerebral embolism are reported in Table 1.

**Table 1:** Demographic, clinical, echocardiographic and microbiologic characteristics of the study population, by presence of cerebral embolism

	Cerebral embolism		p value
	No (N = 425)	Yes (N = 126)	
Age (years, median ± IQR)	69 ± 22	69 ± 19	NS
Female gender (N, %)	140 (33.8%)	45 (36.9%)	NS
BMI (median ± IQR)	24.2 ± 5.2	24.0 ± 4.5	NS
Diabetes (N, %)	81 (19.1%)	23 (18.3%)	NS
Dyslipidemia (N, %)	116 (29.3%)	37 (31.6%)	NS
Hypertension (N, %)	250 (59.1%)	73 (57.9%)	NS
Renal failure (N, %)	101 (23.8%)	31 (24.6%)	NS
Mild	35 (8.2%)	12 (9.5%)	NS
Moderate	38 (8.9%)	12 (9.5%)	
Severe	13 (3.1%)	5 (4.0%)	
Dyalisis	15 (3.5%)	2 (1.6%)	
Cancer (N, %)	95 (22.4%)	22 (17.5%)	NS
PM (N, %)	56 (13.2%)	11 (8.7%)	NS
Oral anticoagulant therapy (N, %)	120 (28%)	40 (32%)	NS
Drug abuse (N, %)	47 (11.1%)	13 (10.3%)	NS
Vegetation length (mm, median ± IQR)	10 ± 11	11 ± 10	0.004
Site of infection			
Aortic valve (N, %)	235 (55.3%)	60 (47.6%)	0.001
Mitral valve (N, %)	149 (35.1%)	64 (50.8%)	
Tricuspid valve (N, %)	41 (9.6%)	2 (1.6%)	
Prosthetic valve (N, %)	172 (40.6%)	50 (39.7%)	NS
Double valve infection (N, %)	69 (16.2%)	23 (18.3%)	NS
Severe valvular dysfunction (N, %)	213 (50.1%)	47 (37.3%)	0.015
Paravalvular extension (N, %)	90 (21.2%)	26 (20.6%)	NS
EF (%,median ± IQR)	58 ± 11	60 ± 10	0.008
TAPSE (mm, median ± IQR)	21 ± 6	19 ± 7	NS
EuroSCORE2 (median ± IQR)	7 ± 12	8 ± 20	NS
Germ (N, %)			
*Streptococci*	69 (16.2%)	20 (15.9%)	0.025
*S. bovis*	37 (8.7%)	4 (3.2%)	
*Staphylococcus aureus*	69 (16.2%)	34 (27.0%)	
*Coagulase negative staphylococci*	56 (13.2%)	12 (9.5%)	
*Enterococci*	88 (20.7%)	18 (14.3%)	
Negative coltures	77 (18.1%)	26 (20.6%)	
Other	29 (6.8%)	12 (9.5%)	

*BMI* body mass index, *PM* pacemaker, *EF* ejection fraction, *TAPSE* tricuspid annular plane systolic excursion

Cerebral embolism was significantly more frequent in patients with large vegetations (p = 0.004), mitral valve infection (p = 0.001), and Staphylococcus aureus infection (p = 0.025). Patients with neurologic complications also had a significantly higher left ventricular ejection fraction and a lower proportion of severe valvular dysfunction. Such a difference is the probable consequence of a referral to a surgical centre since, beyond embolic complications, surgery for IE is indicated by heart failure or severe valvular dysfunction [4]. At multivariable analysis, only vegetation length was an independent predictor of cerebral embolism (HR 1.057, 95% CI 1.025–1.091, p = 0.001), with a best predictive threshold of 10 mm length at ROC analysis (AUC 0.54, p = 0.001).

### Surgical treatment and mortality

Of 551 patients with IE, 431 (78%) underwent surgery, 60 (11%) received only medical therapy because of the absence of surgical indication, and 60 (11%) were excluded from surgery, despite surgical indication, because of prohibitive general conditions.

**Fig. 1:**
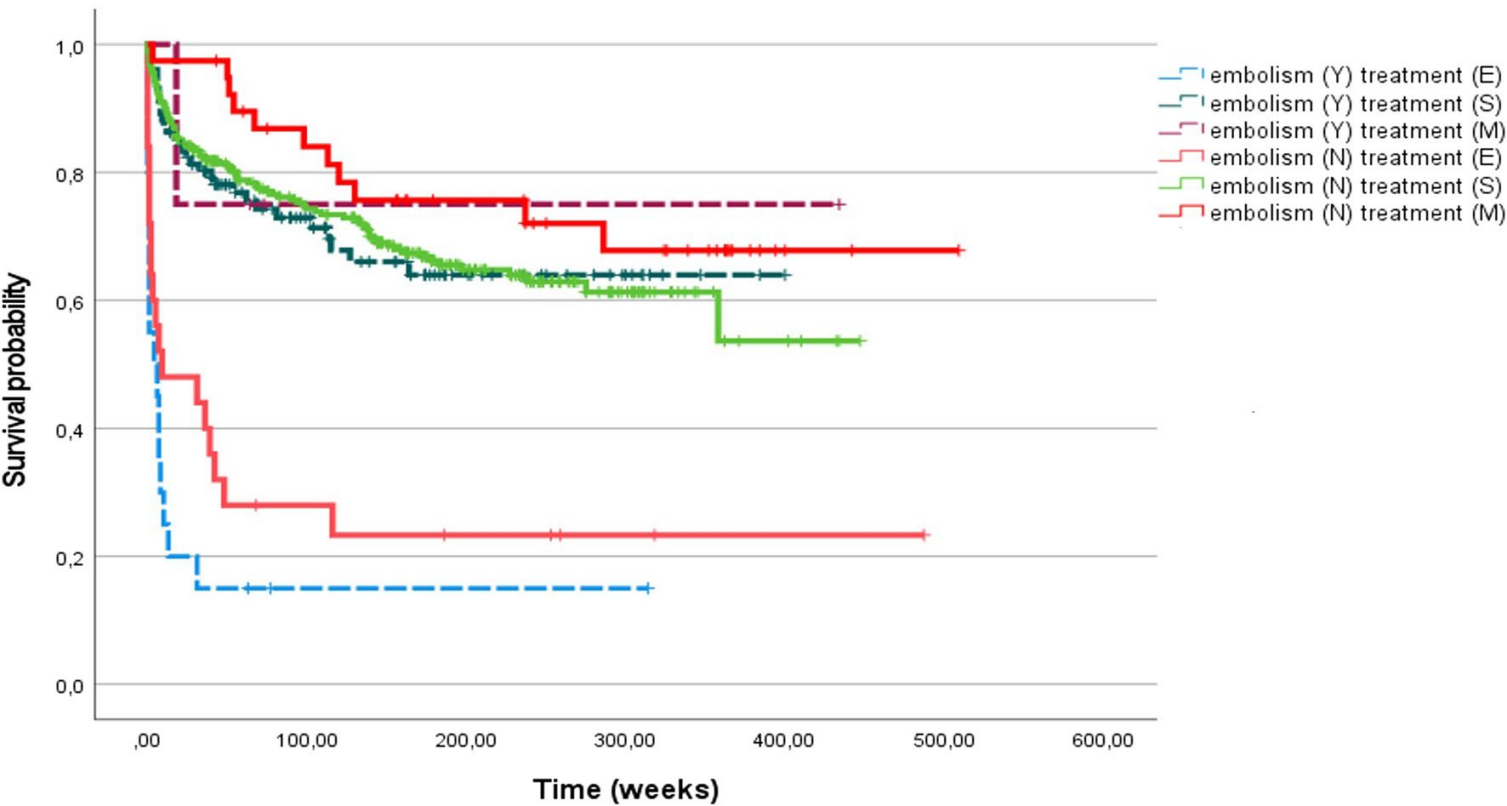
Kaplan–Meier analysis of survival probability of 551 patients with infective endocarditis with (Y) or without (N) cerebral septic embolism, divided for therapeutic strategy: excluded from surgery (E), surgery (S), medical therapy (M)

**Fig. 2:**
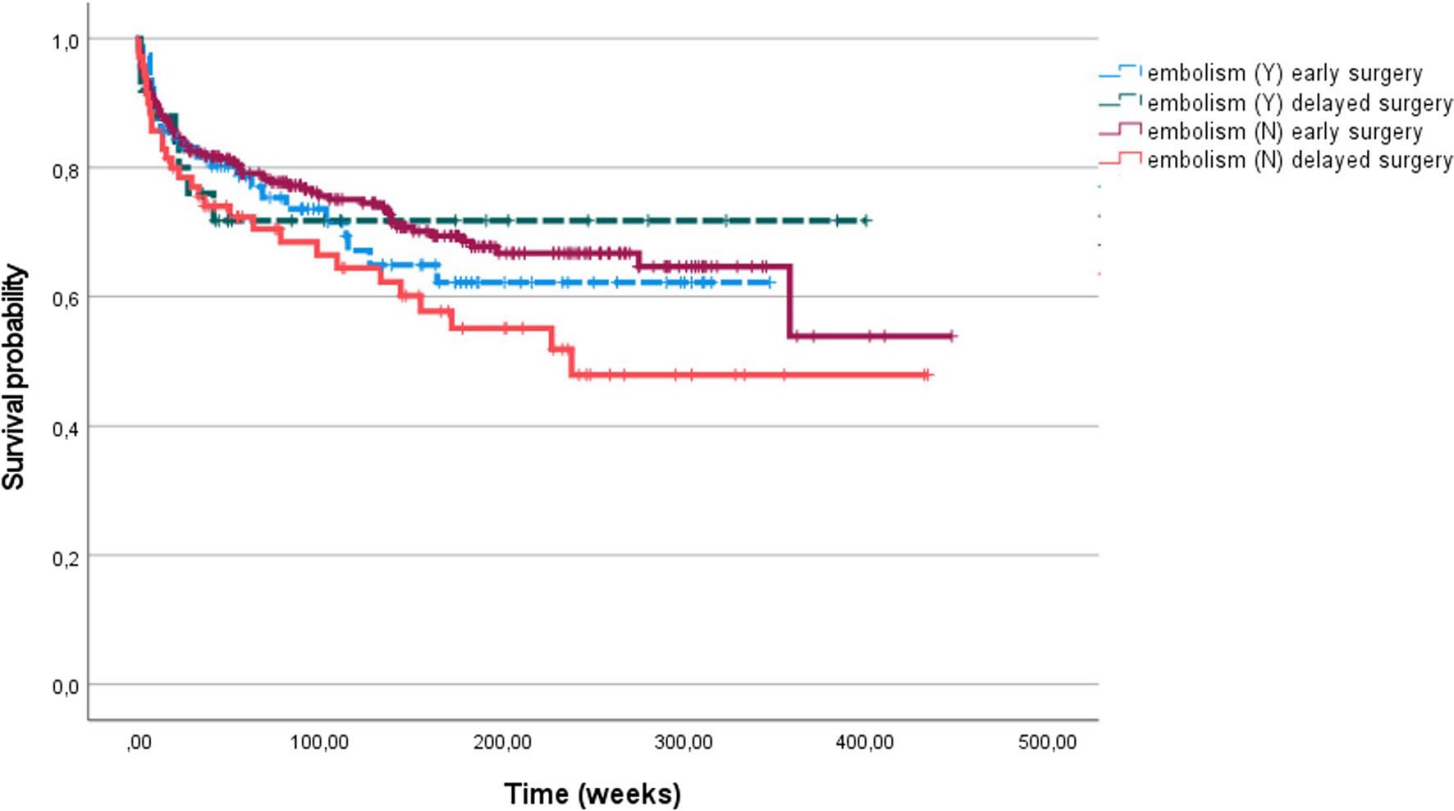
Kaplan–Meier analysis of survival probability of 431 patients with infective endocarditis undergoing cardiac surgery with (Y) or without (N) cerebral septic embolism, divided for therapeutic strategy: early surgery or delayed surgery

Compared to those without, patients with neurologic complications were more often excluded from surgery despite an indication for it (Table 2). As shown in Table 3, severe disability (i.e. Barthel index score class 3) was the main reason for denying surgery. When eligible, patients with or without neurologic complications were operated on within two weeks of IE diagnosis in similar proportions (70% vs 72%) and with similar survival rates for each treatment group (i.e. surgery; medical therapy for the absence of surgical indication; excluded from surgery because of prohibitive general conditions; Fig. 1) and for patients undergoing early surgery (Fig. 2).

**Table 2:** Therapeutic strategies and mortality of patients with infective endocarditis, by presence of cerebral embolism

	Cerebral embolism		p value
	No (N = 425)	Yes (N = 126)	
Treatment (N, %)			
Excluded from surgery despite indication	40 (9.5%)	20 (15.8%)	0.002
Surgery	329 (77.4%)	102 (81.0%)	
No indication for surgery	56 (13.1%)	4 (3.2%)	
Thirty-day mortality (N, %)	36 (8.5%)	13 (10.3%)	NS
Three-year mortality (N, %)	135 (31.8%)	48 (38.1%)	NS

**Table 3:** Clinical and anatomical characteristics of patients with cerebral embolism, by therapeutic strategy

	Treatment			p-value
	Excluded from surgery (N = 20)	Surgery (N = 102)	No indication for surgery (N = 4)	
Age (years, median ± IQR)	73 (66–79)	66 (62–72)	67 (36–78)	NS
Dysability at admission (N, %)				
Mild	4 (21,1%)	56 (62,9%)	3 (75%)	0.001
Moderate	1 (5,3%)	16 (18,0%)	0 (0%)	
Severe	14 (73,7%)	17 (19,1%)	1 (25%)	
Neurologic symptoms (N, %)	18 (90,0%)	69 (75,0%)	2 (50%)	NS
Heamorrhagic cerebral lesions (N, %)	9 (45,0%)	25 (24,5%)	0 (0%)	NS
Site of cerebral lesion (N, %)				
Basal nuclei	1 (5,3%)	4 (4,3%)	1 (25%)	NS
Cerebral lobes	12 (63,2%)	61 (65,6%)	2 (50%)	
Cerebellum	0 (0,0%)	3 (3,2%)	0 (0%)	
Multiple sites	6 (31,6%)	25 (26,9%)	1 (25%)

**Fig. 3:**
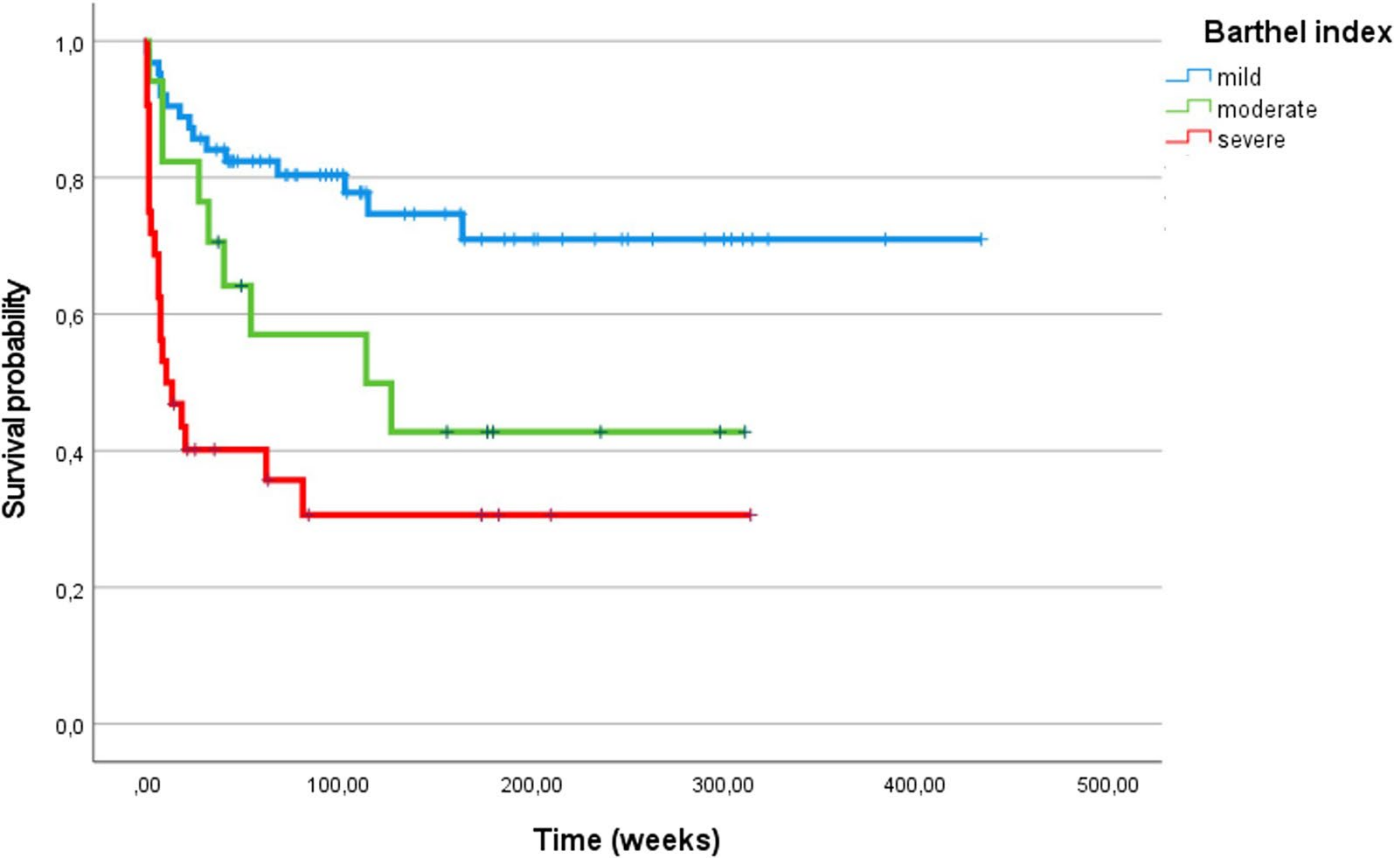
Kaplan–Meier analysis of survival probability of 126 patients with infective endocarditis and cerebral septic embolism, divided for Barthel index, categorized in mild, moderate and severe

**Fig. 4:**
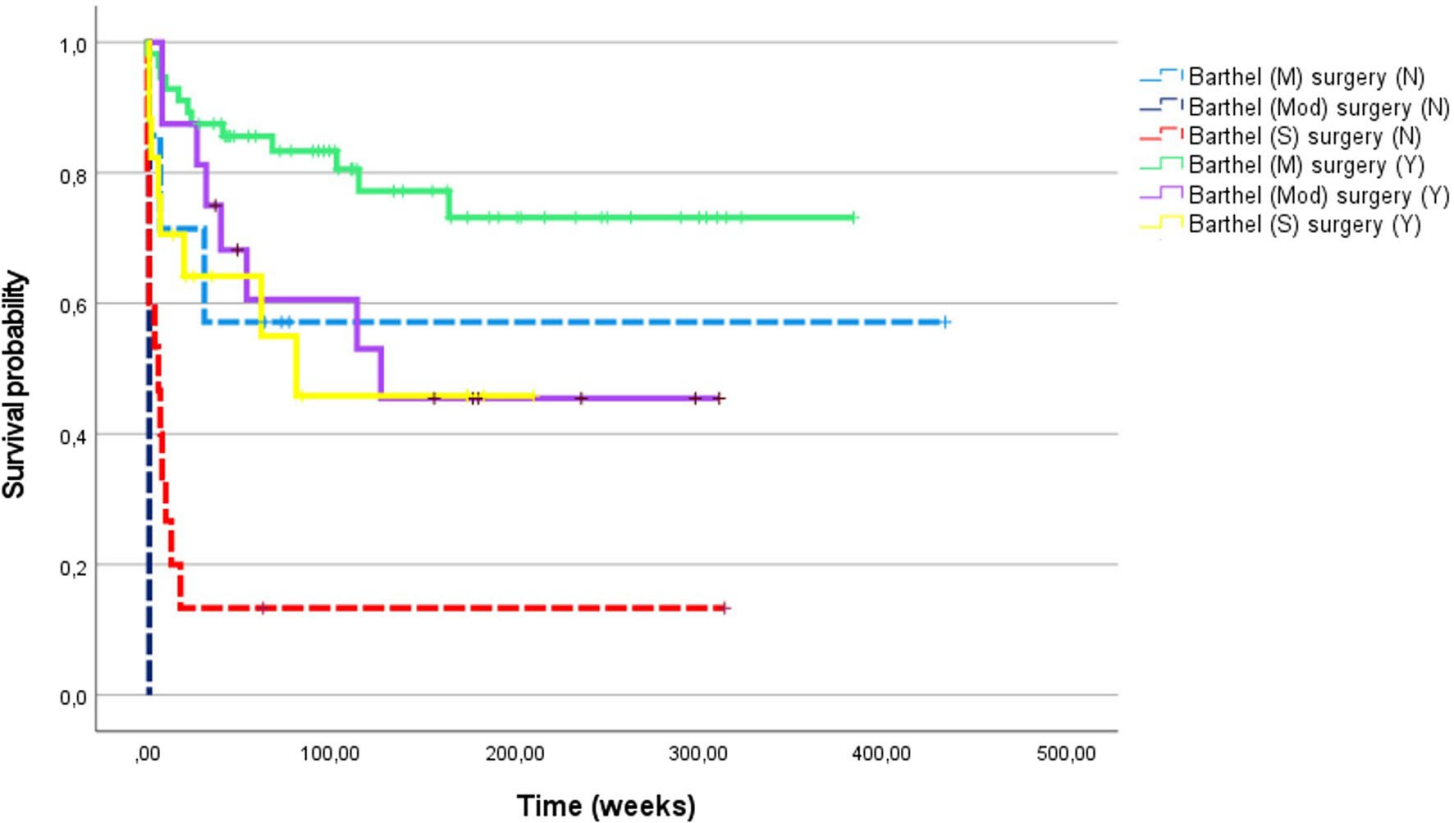
Kaplan–Meier analysis of survival probability of 126 patients with infective endocarditis and cerebral septic embolism, divided for Barthel index, categorized in mild (M), moderate (Mod) and severe (S), and for surgical intervention (Y) or not (N)

Hemorrhagic transformation (p = 0.018), GCS ≤ 14 (p = 0.001) or Barthel index score < 40 (i.e. severe disability; p = 0.001) at presentation were univariate predictors of mortality in IE patients with cerebral embolism. Of these, only the Barthel index latter was kept in the final multivariable model (HR 2.4, 95% CI 1.47–3.95, p = 0.001), together with known adverse prognostic factors of infective endocarditis such as age (HR 1.036 for each increasing year, 95% CI 1.024–1.050), double valve infection (HR 2.27, 95% CI 1.59–3.23), EUROSCORE II (HR 1.015 per unitary increase, 95% CI 1.009–1.021), and exclusion from surgery (HR 4.82, 95% CI 2.42–9.58) [7]. Figure 3 shows the survival curves of patients with cerebral embolism stratified by levels of disability, and Fig. 4 shows the same curves by levels of disability and treatment, highlighting the independent prognostic impact of disability itself.

## Discussion

Our data failed to confirm the independent, negative prognostic impact demonstrated in other clinical series of cerebral embolisms complicating IE [8]. This probably depends on different inclusion criteria since we also enrolled asymptomatic or mildly symptomatic events. Instead, we found that the onset and the severity of functional disability is independently associated with the risk of all-cause death, even after adjusting for a series of instrumental and demographic variables. The value of Barthel index in patients with ischemic stroke has been largely demonstrated [9], while only two studies [5, 10] included it to evaluate patients with neurologic complications of IE. Neither of them, however, used the Barthel index to explore the association of disability with mortality. Besides the anatomical characterization of a cerebral event, the functional status is a major determinant of prognosis and should guide therapeutic strategies. The same neurologic event may hesitate to different levels of disability that, at least in large part, depend on the functional status before the acute event. The Barthel index gives a comprehensive measure of the residual autonomy, which should be considered to avoid futility in terms not only of the quality of life but of survival rate. Conversely, patients with preserved functional status should receive prompt cardiac surgery as indicated.

In accordance with previous studies [3, 5, 10], our data demonstrated the safety of early surgery since patients operated on within two weeks of IE diagnosis had similar survival in the presence or absence of neurologic complications. Interestingly, patients with neurologic complications treated after two weeks had a survival curve similar to that of earlier surgery. This suggests that an individually tailored timing of surgery is the optimal strategy.

Finally, the prevention of embolism has probably the greatest impact on prognosis. We found that a vegetation length greater than 10 mm is an independent predictor of embolism. Guidelines [4] recommend (IIB, level of evidence C) surgery for the primary prevention of embolism, as the sole indication, for vegetations > 15 mm. Considering the potentially devastating consequences of even a single embolic cerebral event and the good results of surgery, we believe that the threshold to indicate surgery should be lowered, especially in patients with a low operative risk and a preserved functional profile as assessed by the Barthel index. Further studies on this issue are necessary.

Our study has some limitations: first, its retrospective nature; second, changes in the clinical management of IE may have occurred during the long study period. Third, it is a real-world single-centre experience. Finally, our study has a potential referral bias since we have conducted it in a high-volume surgical centre; therefore, the percentage of patients with a surgical indication may be regarded as disproportionately high.

## Conclusions

In patients with neurologic complications of IE, the primary determinant of prognosis is the functional status after the event. Early surgery is safe for most patients, but an individually tailored program guarantees a similar prognosis for patients who need delayed surgery.

## Data Availability

The dataset used and analyzed during the current study is available from the corresponding author on reasonable request sharing the SPSS dataset.
